# Glial cell activation, recruitment, and survival of B-lineage cells following MCMV brain infection

**DOI:** 10.1186/s12974-016-0582-y

**Published:** 2016-05-20

**Authors:** James R. Lokensgard, Manohar B. Mutnal, Sujata Prasad, Wen Sheng, Shuxian Hu

**Affiliations:** Neurovirology Laboratory, Department of Medicine, University of Minnesota, 3-220 LRB/MTRF, 2001 6th Street S.E., Minneapolis, MN 55455 USA

## Abstract

**Background:**

Chemokines produced by reactive glia drive migration of immune cells and previous studies from our laboratory have demonstrated that CD19^+^ B cells infiltrate the brain. In this study, in vivo and in vitro experiments investigated the role of reactive glial cells in recruitment and survival of B-lineage cells in response to (murine cytomegalovirus) MCMV infection.

**Methods:**

Flow cytometric analysis was used to assess chemokine receptor expression on brain-infiltrating B cells. Real-time RT-PCR and ELISA were used to measure chemokine levels. Dual-immunohistochemical staining was used to co-localize chemokine production by reactive glia. Primary glial cell cultures and migration assays were used to examine chemokine-mediated recruitment. Astrocyte: B cell co-cultures were used to investigate survival and proliferation.

**Results:**

The chemokine receptors CXCR3, CXCR5, CCR5, and CCR7 were detected on CD19^+^ cells isolated from the brain during MCMV infection. In particular, CXCR3 was found to be elevated on an increasing number of cells over the time course of infection, and it was the primary chemokine receptor expressed at 60 days post infection Quite different expression kinetics were observed for CXCR5, CCR5, and CCR7, which were elevated on the highest number of cells early during infection and decreased by 14, 30, and 60 days post infection Correspondingly, elevated levels of CXCL9, CXCL10, and CXCL13, as well as CCL5, were found within the brains of infected animals, and only low levels of CCL3 and CCL19 were detected. Differential expression of CXCL9/CXCL10 and CXCL13 between microglia and astrocytes was apparent, and B cells moved towards supernatants from MCMV-infected microglia, but not astrocytes. Pretreatment with neutralizing Abs to CXCL9 and CXCL10 inhibited this migration. In contrast, neutralizing Abs to the ligand of CXCR5 (i.e., CXCL13) did not significantly block chemotaxis. Proliferation of brain-infiltrating B cells was detected at 7 days post infection and persisted through the latest time tested (60 days post infection). Finally, astrocytes produce BAFF (B cell activating factor of the TNF family) and promote proliferation of B cells via cell-to-cell contact.

**Conclusions:**

CXCR3 is the primary chemokine receptor on CD19^+^ B cells persisting within the brain, and migration to microglial cell supernatants is mediated through this receptor. Correspondingly, microglial cells produce CXCL9 and CXCL10, but not CXCL13. Reactive astrocytes promote B cell proliferation.

## Background

While it has been well-established that Ab-producing cells of the B-lineage play a local protective role during central nervous system (CNS) infection with encephalitic RNA viruses such as Sindbis virus, Semliki Forest virus, West Nile virus, rabies virus, and neurotropic coronaviruses [1–6]; both the beneficial and detrimental contributions of these lymphocytes within the brain following encephalitis induced by cytomegaloviruses have been largely ignored. We have previously shown that murine cytomegalovirus (MCMV) infection triggers accumulation and persistence of B-lineage cells within the brain, which produce Abs and play a significant role in controlling reactivated virus [7]. While the involvement of chemokines and survival factors in B cell migration and differentiation in lymphoid organs is well-documented, little is known about the glial cell-produced factors which are involved in the recruitment, retention, and long-term survival of these lymphocytes within the brain.

Our previous studies have extensively characterized cytomegalovirus neurotropism both in vitro and in vivo, reviewed in Cheeran et al. [8]. Using primary cell culture systems or brain-derived cell lines, it has been shown that practically all cell types within the brain have some degree of susceptibility to CMV infection. However, these different cell types vary in their ability to support a complete viral replication cycle, which in turn is largely controlled by the transcription factor milieu within the cell during infection. In both mice and humans, cultured primary astrocytes support productive CMV infection with a 3 log_10_ unit increase in viral titers over a course of 5 days. These cells also respond to the virus by producing immune mediators. In contrast to astrocytes, primary differentiated neurons and primary microglial cells are much more refractory to productive CMV replication. Although nonproductively infected, microglial cells are stimulated by viral antigens to produce immune mediators. It is important to distinguish between productive viral infection of glial cells and their innate stimulation by viral antigens through pattern recognition receptors or immune factors. Our previous in vivo studies have shown that subsequent to intracerebroventricular (icv) infection with MCMV, in immunocompetent animals, viral brain infection is localized primarily to cells that line the periventricular region. These periventricular target cells were subsequently identified as nestin-positive, neural stem cells [9]. Infection spreads to astrocytes within the brain parenchyma only in the absence of an effective CD8^+^ T cell response [10]. Reports by other groups have also established the importance of CD8^+^ T cells for control of primary infection [11, 12]. Likewise, previous studies from our laboratory have shown that antigen-specific CD8^+^ T cells persist within the brain even in the absence of detectable viral protein [13]. Establishment of latency after clearance of acute infection and the potential to reactivate to recurrent infection are key features of all herpesvirus, including MCMV [14].

During normal development of high-affinity Ab-secreting cells, germinal center plasma blasts give rise to long-lived plasma cells which reside primarily in the bone marrow. The bone marrow niche provides factors necessary to support long-term survival of these cells in order to maintain serum Ab levels and protect against re-infection [15, 16]. Because passage of Abs from serum into the brain through an intact blood–brain barrier is inefficient, local Ab secretion by cells which have infiltrated the brain appears to be a more effective strategy for viral control. Cells of the B-lineage enter the CNS in response to acute viral infection, and like T cells, they are retained within the brain into the chronic phase. Although CD8^+^ T cells play a critical role in controlling viral spread during acute brain infection [10, 11], prolonged immune responses within the brain following MCMV infection are characterized by persistence of Ab-producing B cells, chronic microglial cell activation, and retention of virus-specific memory CD8^+^ T cells [7, 13]. Like other herpesviruses, MCMV establishes latency after control of acute infection and clearance of detectable viral antigen. We have previously found that Abs produced within the CNS play a significant role in controlling MCMV reactivation from the latent state [7].

A number of chemokines and their receptors have been demonstrated to regulate B cell responses in lymphoid organs [17]. The chemokine receptor CXCR5 (and its ligand CXCL13), as well as CCR7 (and its ligands CCL19 and CCL21), has been shown to play important roles in trafficking of B cells to lymphoid follicles in the development of germinal centers. Specifically within the CNS, during multiple sclerosis, but not viral infection, CXCL13 has been associated with formation of ectopic follicle-like structures and recapitulation of all stages of B cell differentiation observed in secondary lymphoid organs [18, 19]. It has also been shown to be a major chemokine receptor for B cell recruitment to the CNS during several neuroinflammatory diseases in patients [20]. The chemokine receptor CXCR3 is normally absent on naïve B cells, but it is upregulated during differentiation into memory and plasma cell precursors [21]. Furthermore, in knockout animals, the absence of CXCR3 has been demonstrated to impair recruitment of Ab-secreting cells into the CNS in a glia-tropic mouse hepatitis virus (MHV)-JHM model [22]. Using a Sindbis virus infection model, CCR5 was detected on 42 % of CD19^+^ B cells within the infected CNS, suggesting this receptor may also have recruiting functions [23].

In the present study, using both in vivo and in vitro experiments, we examined the production of known B cell-attracting chemokines by reactive glial cells and examined their role in driving B lymphocyte infiltration and persistence within the brain in response to MCMV infection. We also examined the role of activated glia in promoting B cell survival and proliferation.

## Methods

### Ethical approval

This study was carried out in strict accordance with recommendations in the Guide for Care and Use of Laboratory Animals of the National Institutes of Health. The protocol was approved by the Institutional Animal care and Use Committee (Protocol Number: 140231307A) of the University of Minnesota.

### Virus

RM461, a MCMV expressing *Escherichia coli* β-galactosidase under the control of the human ie1/ie2 promoter/enhancer [24], was kindly provided by Edward S. Mocarski. The virus was maintained by passage in weanling female Balb/c mice (Charles River, Wilmington, MA). Salivary gland-passed virus was then grown in NIH 3T3 cells for two passages, which minimized any carry-over of salivary gland tissue. Infected 3T3 cultures were harvested at 80 to 100 % cytopathic effect and subjected to three freeze–thaw cycles. Cellular debris was removed by centrifugation (1000×*g*) at 4 °C, and the virus was pelleted through a 35 % sucrose cushion (in Tris-buffered saline (TBS), 50 mM Tris–HCl, 150 mM NaCl, pH 7.4) at 23,000×*g* for 2 h at 4 °C. The pellet was suspended in TBS containing 10 % heat-inactivated fetal bovine serum (FBS). Viral stock titers were determined on 3T3 cells as 50 % tissue culture infective doses (TCID_50_) per milliliter.

### Intracerebroventricular infection of mice

Infection of mice with MCMV was performed as previously described [25]. Briefly, female C57BL/6 mice (8 weeks old) were anesthetized using a combination of ketamine and xylazine (100 mg and 10 mg/kg body weight, respectively) and immobilized on a small animal stereotactic instrument equipped with a Cunningham mouse adapter (Stoelting Co., Wood Dale, IL). The skin and underlying connective tissue were reflected to expose reference sutures (sagittal and coronal) on the skull. The sagittal plane was adjusted such that the bregma and lambda were positioned at the same coordinates on the vertical plane. Virulent, salivary gland-passaged MCMV RM461 (1 × 10^5^ TCID_50_ units in 10 μl) was injected into the right lateral ventricle at 0.9 mm lateral, 0.5 mm caudal, and 3.0 mm ventral to the bregma using a Hamilton syringe (10 μl) fitted to a 27 G needle. The injection was delivered over a period of 3–5 min. The opening in the skull was sealed with bone wax, and the skin was closed using 4-0 silk sutures.

### Isolation of brain leukocytes and flow cytometry

Leukocytes were isolated from the brains of the MCMV-infected C57BL/6 mice using a previously described procedure with minor modifications [26–28]. In brief, the whole brains were harvested, pooled (*n* = 2–4 animals/group/experiment), minced finely in RPMI 1640 (2 g/l D-glucose and 10 mM HEPES), and digested in 0.0625 % trypsin (in Ca/Mg-free Hanks’ balanced salt solution (HBSS)) at room temperature for 20 min. Single-cell preparations from the brains were suspended in 30 % Percoll and banded on a 70 % Percoll cushion at 900×*g* at 4 °C for 15 min. Brain leukocytes obtained from the 30–70 % Percoll interface were collected and used for subsequent Ab staining for flow cytometry. For Ab staining, the brain leukocytes were first treated with Fc block (anti-CD32/CD16 in the form of 2.4G2 hybridoma culture supernatant with 2 % normal rat and 2 % normal mouse serum) to inhibit nonspecific Ab binding and were stained with anti-mouse cell surface markers for 45 min at 4 °C (anti-CD45-PE-Cy5, anti-CD11b-AF700, anti-CD19-FITC, anti-Ki67-APC, and anti-CD267(TACI)-PE (eBiosciences, San Diego, CA); and anti-CD269(BCMA)-FITC (R&D Systems, Minneapolis, MN)). Analysis by flow cytometry was performed. Control isotype Abs were used for all fluorochrome combinations to assess nonspecific Ab binding. Live leukocytes were gated using forward scatter and side scatter parameters on a BD FACSCanto flow cytometer (BD Biosciences, San Jose, CA). Data was analyzed using FlowJo software (FlowJo, Ashland, OR).

### Immunohistochemistry

The brains were harvested from animals that had been perfused with a series of phosphate-buffered saline (PBS), 2 % sodium nitrate, and 4 % paraformaldehyde. The murine brains were subsequently submerged in 4 % paraformaldehyde for 24 h and transferred to 25 % sucrose solution for 2 days prior to sectioning. After blocking (10 % normal goat serum and 0.3 % Triton X-100 in PBS) for 1 h at room temperature (RT), the brain sections (25 μm) were incubated overnight at 4 °C with the following primary Abs: rat anti-mouse CD3 (10 μg/ml; R&D Systems, Minneapolis, MN) and rat anti-mouse CD19 (15 μg/ml; Biolegend, San Diego, CA). After washing three times with TBS, secondary Ab (goat anti-rat IgG biotinylated; Vector Labs, Burlingame, CA) was added for 1 h at RT followed by incubation with ABC (avidin-biotinylated enzyme complex, Vector Labs) solution. The peroxidase detection reaction was carried out using 3,3′-diaminobenzidine tetrahydrochloride (DAB; Vector Labs) for several minutes at RT. Double immunolabeling was performed using secondary goat anti-rat HRP-conjugated Ab (Vector Labs) and developed with HistoGreen substrate (Linaris, Dossenheim, Germany). For double immunofluorescence staining, goat anti-mouse CXCL10 (15 μg/ml), CXCL13 (10 μg/ml), and goat anti-BAFF (B cell activating factor of the TNF family, 15 μg/ml) Abs (R&D Systems) and rabbit anti-GFAP (glial fibrillary acidic protein, 1:500 dilution; DAKO, Carpenteria, CA) and rabbit anti-Iba1 (ionized calcium-binding adaptor molecule 1) (1 μg/ml; Wako Chemicals, Richmond, VA) Abs were used followed by donkey anti-goat Alexa Fluor 488 and donkey anti-rabbit Alexa Fluor 594 Abs with nuclear labeling using Hoechst 33342 (1 μg/ml; Chemicon, Temecula, CA) and viewed under a fluorescent microscope.

### Real-time PCR

RNA from the brain tissue was extracted using TRIzol reagent (Invitrogen, Carlsbad, CA), respectively. cDNA was synthesized with 1.0 μg of total RNA using Superscript III reverse transcriptase (Invitrogen) and oligo d(T)_12–18_ primers (Gene Link, Hawthorne, NY). PCR was performed with the SYBR Advantage qPCR master mix (ClonTech, Mountain View, CA). The PCR conditions for the Mx3000P QPCR System (Stratagene, now Agilent Technologies, La Jolla, CA) were as follows: 1 denaturation cycle at 95 °C for 10 s; 40 amplification cycles of 95 °C for 10 s, 60 °C annealing for 10 s, and elongation at 72 °C for 10 s, followed by 1 dissociation cycle. The relative product levels were quantified using the 2^−∆∆Ct^ method [29] and were normalized to the housekeeping gene hypoxanthine phosphoribosyl transferase (HPRT).

### Enzyme-linked immunosorbent assay

The murine brains were homogenized in DMEM containing 1 % FBS and were centrifuged at 4 °C for 15 min to harvest supernatants used in enzyme-linked immunosorbent assay (ELISA). Protein concentrations were measured using a Bradford assay (Bio-Rad, Hercules, CA). The supernatants from MCMV- and cytokine-stimulated astrocyte and microglial cultures (48 h) were collected for ELISA. In brief, 96-well ELISA plates pre-coated with anti-mouse CXCL9, CXCL10, CXCL13, CCL3, CCL5, CCL19, or BAFF Abs (2 μg/ml) overnight at 4 °C were blocked with 1 % BSA in PBS for 1 h at 37 °C. After washing (PBS with Tween 20), the supernatants and a series of diluted standards were added to the wells for 2 h at 37 °C. Detection Abs of anti-CXCL9, CXCL10, CXCL13, CCL3, CCL5, CCL19, or BAFF were added for 90 min at 37 °C followed by addition of secondary Abs conjugated with horseradish peroxidase (1:10,000) for 45 min at 37 °C. The chromogen substrate K-Blue (Neogen, Lexington, KY) was added for color development which was terminated with 1 M H_2_SO_4_. The plates were read at 450 nm, and chemokine levels were extrapolated from standard curves and normalized to protein concentrations.

### B cell isolation

The spleens from MCMV-primed (RM461, 1 × 10^4^ TCID_50_, i.p.) donor animals were collected aseptically at 7 days post-priming. Single-cell suspensions were depleted of RBC through treatment with 0.87 % ammonium chloride, washed twice, and cell viability was confirmed using trypan blue. B lymphocytes were enriched by negative selection using a MagCellect isolation kit following the manufacturer’s instructions (R&D Systems). Isolated B cells were consistently >99 % CD19^+^ as evaluated by flow cytometry and had a viability of 99 % as evaluated by trypan blue dye exclusion.

### Chemotaxis assay

B cell migration assays were performed using a 96-well cell migration system (Neuro Probe, Gaithersburg, MD) with 5-μm pore size polycarbonate membrane filters. The isolated B cells suspended in DMEM containing 2 % FBS were loaded onto the upper chambers (2 × 10^5^ cells in 100 μl per well). The lower chambers were filled with 300 μl of media with or without recombinant chemokines or glial cell-conditioned media. After incubation for 4 h at 37 °C, migrated cells in the lower chamber were either collected and counted at a high flow rate for 1 min using flow cytometry or AlamarBlue dye (10 μl) was added to the lower chamber, incubated overnight at 37 °C, and read with a fluorescent plater reader (Ext_544 nm_/Em_590 nm_) to assess the number of migrated cells. All assays were performed in triplicates or quadruplicates. In the blocking experiments, neutralizing Abs to chemokines were pre-incubated with glial cell-conditioned media for 30 min at 37 °C prior to being loaded into the chambers.

### Primary glial cell cultures

Murine cerebral cortical cells from 1-day-old mice were dissociated after a 30-min trypsinization (0.25 %) in HBSS and plated in 75 cm^2^ culture flasks in DMEM containing 6 % FBS, penicillin (100 U/ml), streptomycin (100 μg/ml), gentamicin (50 μg/ml), and Fungizone® amphotericin B (250 pg/ml). The medium was replenished 1 and 4 days after plating. On day 12 of culture, floating microglial cells were harvested and plated onto 48-well cell culture plates (1 × 10^5^ cells/well). After a 1-h incubation at 37 °C, the culture plates were washed and incubated overnight before starting the experiments. Purified microglial cell cultures were comprised of a cell population in which >95 % stained positively with Iba-1 antibodies and 3–5 % stained positively with antibodies specific to GFAP. Purified astrocyte cultures were prepared from the culture flask following isolation of microglia at 14 days in vitro. Briefly, after collection of microglia, the culture flasks were shaken at 180–200 rpm at 37 °C for 16 h followed by trypsinization (0.25 % trypsin in HBSS) for 30 min. After adding FBS (final concentration 10 %), centrifugation, and washing, the cells were seeded into new flasks with DMEM followed by a medium change after 24 h. The subculture procedure was repeated weekly for 2–3 times to remove residual oligodendrocytes and microglia in order to achieve highly purified astrocyte cultures (95–98 % of cells reacted with GFAP Ab, 3–5 % stained with Iba-1 Ab), which were plated onto 48-well culture plates (1 × 10^5^ cells/well).

### Statistical analysis

Pooled data are presented as mean (±SEM) derived from independent experiments. Representative data are presented as mean (±SD) of replicate samples. All statistical analysis was performed using analysis of variance (ANOVA) followed by Scheffe post hoc test.

## Results

### B cells are recruited into the brain and persist following MCMV infection

**Fig. 1:**
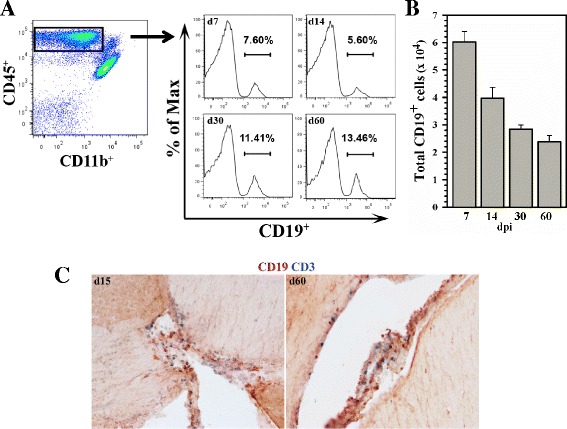
Long-term persistence of CD19^+^ B cells within the CNS following MCMV brain infection. Single-cell suspensions of brain tissue obtained from MCMV-infected mice were banded on a 70 % Percoll cushion. Brain leukocytes were collected and labeled with PE-Cy5-conjugated Abs specific for CD45, AF700-labeled anti-CD11b, PE-Cy7 anti-CD3, and Cy7-APC anti-CD19 and analyzed using flow cytometry. **a** Representative histograms show the percentage of CD45^+^CD19^+^ cells within the brain-infiltrating CD45^hi^CD11b^lo^CD3^−^ population obtained from animals at 7, 14, 30, and 60 days post infection **b** Anti-CD19 staining was used to determine the total number of B cells within the infiltrating CD45^hi^ CD3^−^ population. Pooled data are presented as mean (±SEM) absolute number of infiltrating cells from three independent experiments (*n* = 6–7 animals/time point). **c** Immunohistochemical staining demonstrating persistence of CD19^+^ B cells (*brown*), as well as CD3^+^ T cells (*blue*) within infected brains at 15 and 60 d.p.i.

Previous studies from our laboratory have identified CD19^+^ B lymphocytes within the CD45^hi^CD11b^lo^ leukocyte population infiltrating the brain in response to MCMV infection [7, 30]. In this study, multi-color flow cytometry was used to investigate the recruitment, persistence, and survival of this leukocyte population. Data generated from these experiments showed that CD19^+^ B cells entered the brain early during the course of infection (i.e., by 7 days post infection) and persisted through at least 60 days post infection, the latest time point tested. At 60 days post infection, 13.46 % of the CD45^hi^CD11b^lo^ leukocytes isolated from the infected brains were CD19^+^ (Fig. 1a). The absolute number of CD19^+^ cells was calculated from the flow cytometry data (Fig. 1b). Results presented in this study were obtained using MCMV infection of C57BL/6 mice, which differs from MCMV infection of the BALB/c animals used in our previous study [30]. We have directly compared the B cell brain infiltration and retention kinetics between these mouse strains. In these studies, the MCMV-infected BALB/c mice had higher, increasing levels of CNS B cells over the time course of infection. In contrast, the C57BL/6 animals had lower, decreasing CNS B cell levels. The results from these experiments showed 2.5 × 10^5^ CD19^+^ B cells within the brain of the BALB/c animals at 30 days post infection, which is the same level as reported in Mutnal et al. [30]. In contrast, in the present study, we found 2.8 × 10^4^ CD19^+^ B cells within the brains of the C57BL/6 animals at 30 days post infection (Fig. 1b). We do not yet know why these differences exist between the two mouse strains, but MCMV infection is clearly much more robust in the BALB/c animals. It is important to point out that the efficiency of lymphocyte extraction from all tissues, including the brain, for flow cytometry may be considerably less than 100 % [31].

B cell presence within the CNS following viral infection was further analyzed using immunohistochemical staining. Thin sections of the brain tissue obtained 15 and 60 days post infection and stained using Mabs against CD19 confirmed the presence of these cells which were localized in the periventricular region (Fig. 1c). Double-immunostaining using anti-CD3 along with anti-CD19 Mabs also demonstrated the presence of T cells within this region (Fig. 1c).

### Chemokine receptors are differentially expressed on brain-infiltrating B cells

**Fig. 2:**
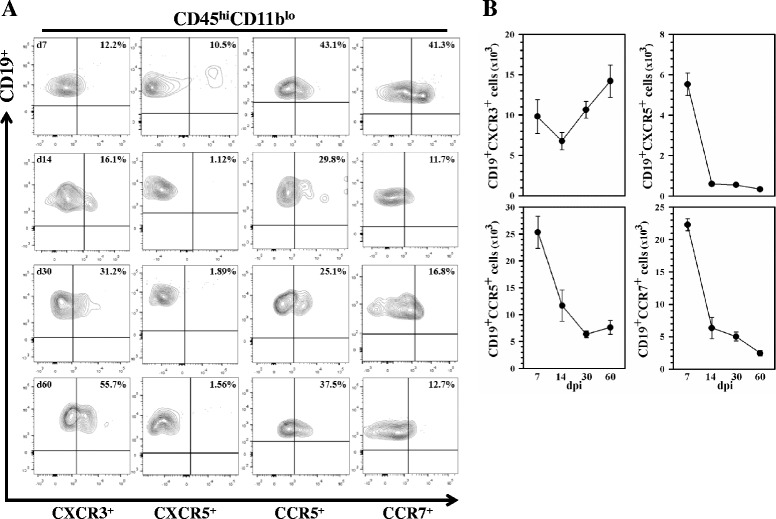
Chemokine receptor expression on brain-infiltrating CD19^+^ B cells. **a** Representative contour plots show the percentage of CD19^+^ B cells isolated from the brains expressing the CXCR3, CXCR5, CCR5, and CCR7 chemokine receptors as assessed using flow cytometry at 7, 14, 30, and 60 increasing proportion of B cells as time **b** Line graphs display the total number (±SEM) of CD19^+^CXCR3^+^, CD19^+^CXCR5^+^, CD19^+^CCR5^+^, and CD19^+^CCR7^+^ B cells within the brain-infiltrating leukocyte population pooled from two independent experiments (*n* = 4–5 animals/time point) at 7, 14, 30, and 60 increasing proportion of B cells as time

B cells are known to express select chemokine receptors which may be involved in homing to MCMV-infected brain. Therefore, levels of CXCR3 (receptor for CXCL9 and CXCL10), CXCR5 (receptor for CXCL13), CCR5 (receptor for CCL3 and CCL5), and CCR7 (receptor for CCL19) were analyzed on CD45^hi^CD11b^lo^CD19^+^ B cells which infiltrated the brain at 7, 14, 30, and 60 days post infection. All of these known B cell chemokine receptors were detected on CD19^+^ cells isolated from the brain during MCMV infection (Fig. 2a). In particular, CXCR3 was found to be elevated on an increasing number of cells over the time course of infection and it was the primary chemokine receptor expressed at 60 days post infection (Fig. 2b). Quite different expression kinetics were observed for CXCR5, CCR5, and CCR7, which were elevated on the highest number of cells early during infection and decreased by 14, 30, and 60 days post infection (Fig. 2b). These results suggest that the CXCR3 ligands (CXCL9 and CXCL10) may be major contributors to B cell recruitment and persistence within the brain following MCMV infection. However, the expression level of a particular chemokine receptor alone may not be a good indicator of its importance in migration, as other factors such as chemokine levels and ligand affinities are also involved. However, CXCR3 was the only chemokine receptor which was found on an increasing proportion of B cells as time p.i. progressed (Fig. 2b). In addition to triggering cell migration into the brain, CXCR3 expression may provide additional B cell maturation and survival functions [32], which may become more apparent at the later time points as infection progresses.

### B cell-attracting chemokines are produced within infected brains

**Fig. 3:**
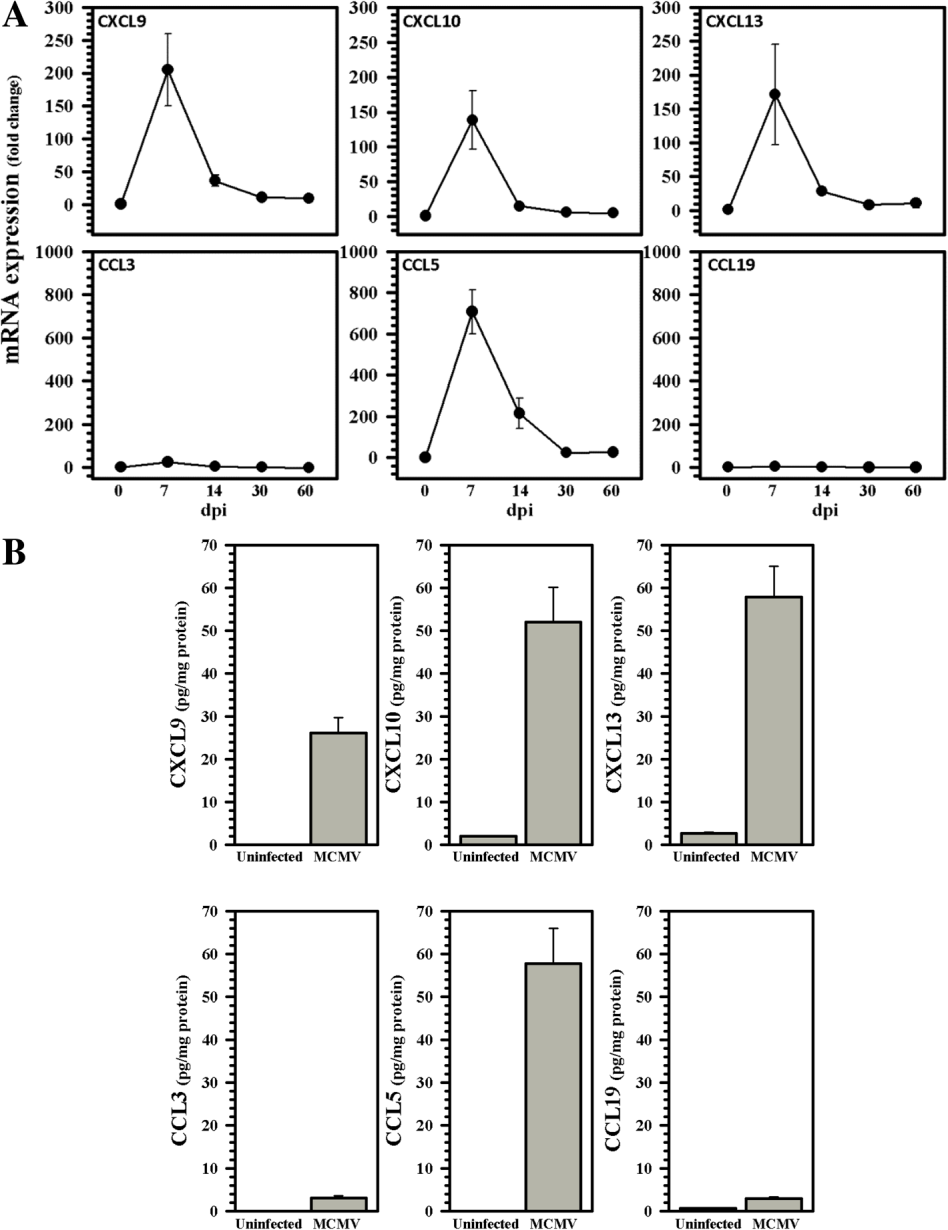
Expression of relevant B cell chemotactic factors within MCMV-infected brains. **a** Expression of chemokine mRNAs relevant to B cell migration was detected in a brain using real-time RT-PCR over the indicated time course of infection. Data are expressed as mean ± SEM from two separate experiments using two animals/time point. **b** The levels of select chemokine proteins within MCMV-infected (MCMV) brain homogenates were quantified at 7 increasing proportion of B cells as time using ELISA. Data presented are mean ± SEM from two separate experiments using three animals/group

To characterize the chemokines expressed within the brain following MCMV infection, chemokine messenger RNAs (mRNAs) were assessed by real-time RT-PCR. The elevated levels of CCL5, CXCL9, CXCL10, and CXCL13 transcripts were detected from the infected brains (Fig. 3a). All of the upregulated chemokine mRNAs reached peak levels at 7 increasing proportion of B cells as time and declined substantially at 14 increasing proportion of B cells as time. The corresponding protein levels of CXCL9, CXCL10, and CXCL13, as well as CCL5, were also found to be elevated using ELISA at 7 increasing proportion of B cells as time (Fig. 3b). In contrast, low levels of CCL3 and CCL19 mRNA were found within the brains of the infected animals, and only minimal protein levels were detectable using ELISA (Fig. 3a, b).

### Chemokines are co-localized with reactive glia in vivo

**Fig. 4:**
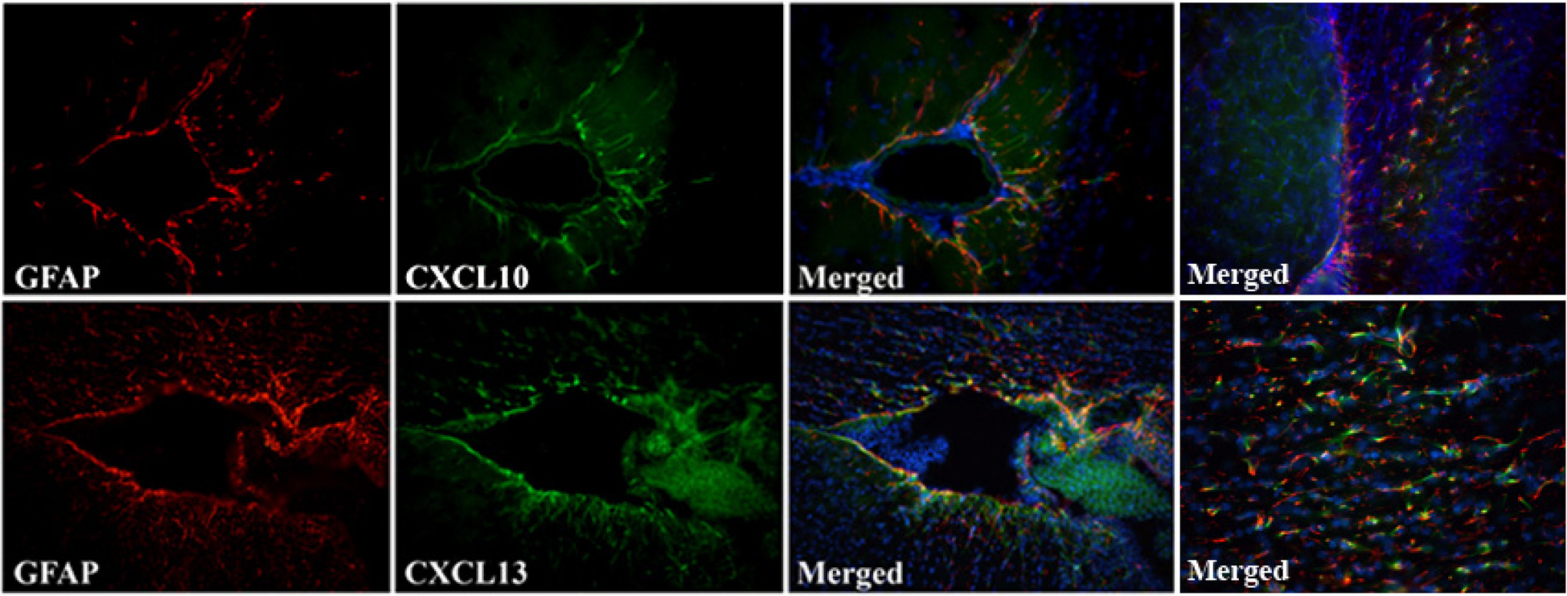
Immunohistochemical staining for chemokine production by reactive glia. Twenty-five-micrometer sections of the brain tissue obtained from MCMV-infected animals at 30 increasing proportion of B cells as time were immunostained using Abs against *CXCL10* (*green*) and *CXCL13* (*green*) along with *GFAP* (*red*) to identify astrocytes producing these chemokines in vivo*. Merged* images demonstrate chemokine production localized to astrocytes

To determine the cellular distribution of CXCL10 and CXCL13 production, we performed dual-immunofluorescence staining for these chemokines along with specific glial cell markers on brain tissue sections obtained from animals at 30 increasing proportion of B cells as time. In these studies, CXCL10 and CXCL13 were both found to co-localize with GFAP-expressing astrocytes (Fig. 4).

### Reactive glial cell cultures produce chemokines

**Fig. 5:**
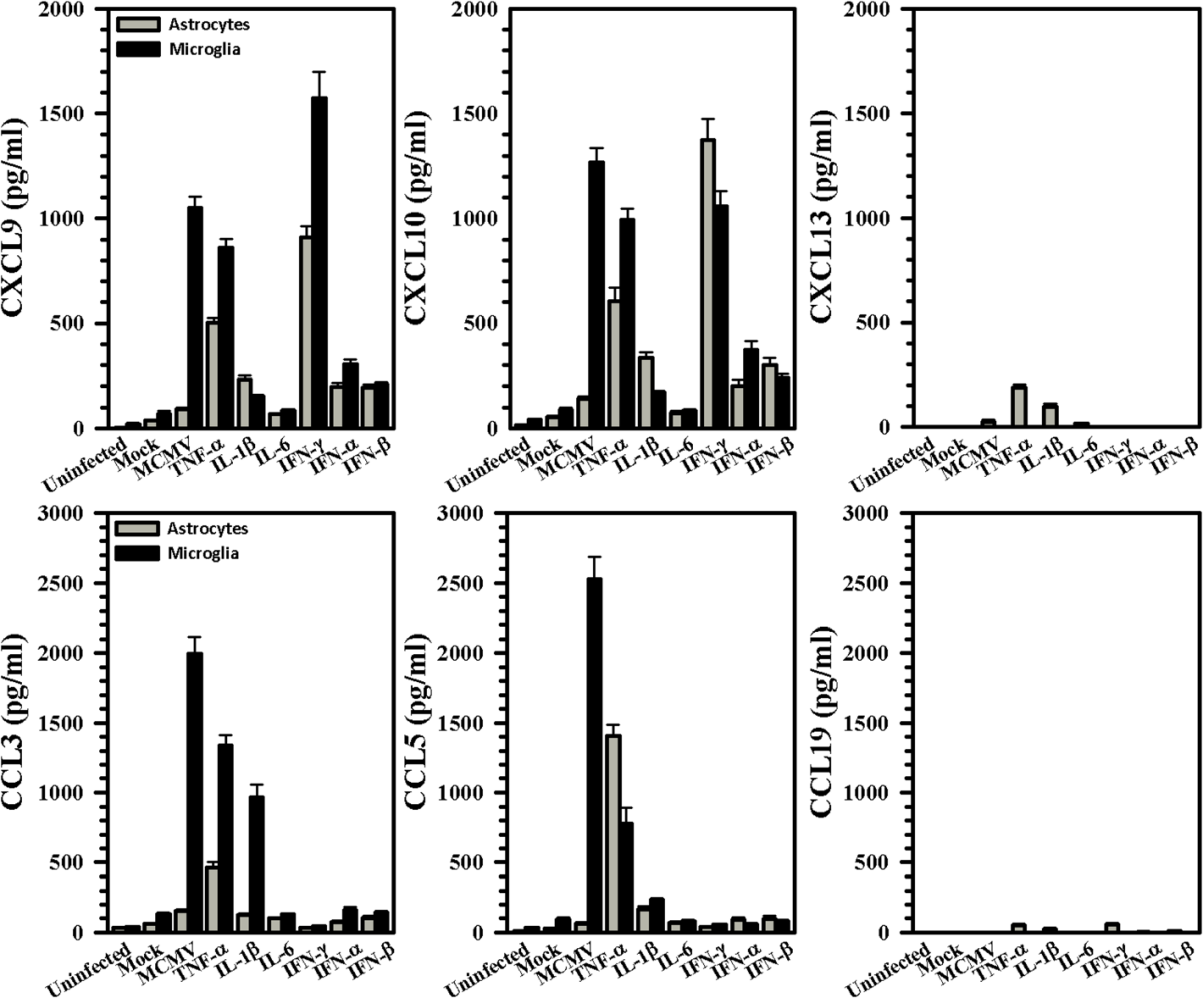
Production of B cell chemotactic factors following viral infection and cytokine stimulation of cultured primary murine microglial cells and astrocytes. Primary murine microglial cell (*black bars*) or astrocyte (*gray bars*) cultures in 48-well plates (1 × 10^5^ cells/well) were infected with MCMV (MOI = 5) or stimulated with the indicated pro-inflammatory cytokine (i.e., TNF-α and IL-6, 20 ng/ml; IL-1β and IFN-γ, 10 ng/ml; IFN-α and IFN-β, 200 U/ml) for 48 h. Production of select B cell-attracting chemokines was assessed using ELISA. Pooled data are presented as mean ± SEM from three to four independent experiments

Production of select B cell-attracting chemokines by primary murine microglial cell or astrocyte cultures stimulated by viral infection (multiplicity of infection (MOI) = 5) or pro-inflammatory cytokine treatment was assessed using ELISA at 48 h post-stimulation. Although cultured astrocytes do support productive viral infection, it is important to distinguish between MCMV infection of glial cells and their innate stimulation by viral antigens through pattern recognition receptors or immune factors which occur at the early time points examined. In these experiments, differential production of CXCL9/CXCL10 and CXCL13 by stimulated glial cells activated in response to MCMV infection was apparent. Microglial cells were found to produce high levels of CXCL9 and CXCL10 in response to viral infection, while infected astrocyte cultures produced markedly lower levels (Fig. 5). Surprisingly, very low level production of CXCL13 (as well as CCL19) was detected in astrocyte cultures stimulated with TNF-α and IL-1β, but not in response to the virus itself (Fig. 5). Viral infection also stimulated high levels of CCL3 and CCL5 production from microglia, but not from astrocyte cultures.

### B lymphocytes migrate towards glial cell culture supernatants

**Fig. 6:**
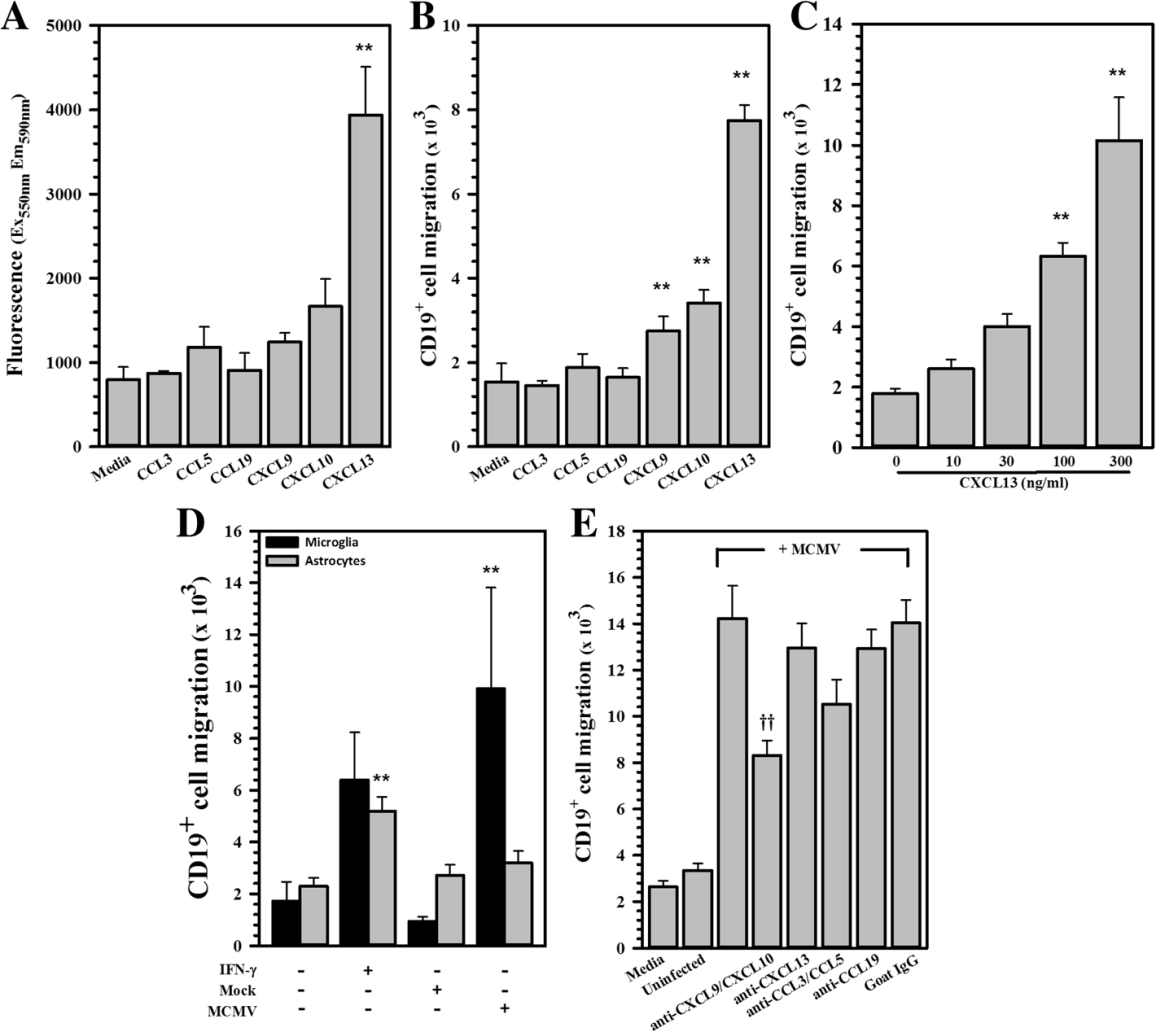
B cell migration towards recombinant chemokines and supernatants from stimulated glial cell cultures. CD19^+^ cells were isolated from the spleens of the MCMV-primed mice (7 days), and migration was assessed using chemotaxis chambers. **a** Migration of B cells towards select recombinant chemokines (100 ng/ml; 300 ng/ml for CXCL13). After 4 h, the migrated cells were stained using Alamar blue and read with a fluorescent plate reader (Ex_550 nm_ Em_590 nm_). **p < 0.0001 vs. media alone. **b** Total numbers of migrated CD19^+^ cells were determined using flow cytometry. Data (mean ± SD) presented are representative of three independent experiments. **p = 0.0034 for CXCL9 and **p < 0.0001 for CXCL10 and CXCL13 vs. media alone. **c** B cell migration to recombinant CXCL13 (dose 0 to 300 ng/ml). Pooled data are (mean ± SEM) derived from two independent experiments. **p = 0.0038 for 100 ng/ml and **p < 0.0001 for 300 ng/ml vs. 0 ng/ml. **d** Migration of B cells towards culture supernatants obtained from IFN-γ-stimulated (10 ng/ml) or MCMV-infected (MOI = 5) primary microglial cell and primary astrocyte cultures at 48 h post-stimulation or infection. B cell migration was assessed after 4 h by flow cytometry. Data (mean ± SD) shown are representative of two separate experiments. **p < 0.0001 for astrocytes vs. untreated and **p = 0.0044 for microglia vs. untreated. **e** Blockade of specific migration towards MCMV-infected (MOI = 5) microglial cell supernatants. Anti-chemokine Abs were added (10 μg/ml) to supernatants from infected microglial cell cultures for 1 h prior to being loaded onto the chemotaxis chamber for assessment of effects on migration. B cell migration was assessed after 4 h by flow cytometry. Pooled data are (mean ± SEM) derived from two independent experiments. ^††^ *p* = 0.0083 vs. MCMV alone

Since chemokines produced by reactive glial cells during viral infection likely serve as signals for leukocyte chemotaxis into the brain, we went on to assess the ability of B cells to migrate. We first determined that B cells obtained from MCMV-primed mice were competent to move towards the recombinant chemokines CXCL9 (100 ng/ml), CXCL10 (100 ng/ml), and CXCL13 (300 ng/ml) (Fig. 6a and b). Interestingly, no significant B cell migration to recombinant CCL3, CCL5, or CCL19 (each at 100 ng/ml) was observed (Fig. 6a and b). A dose–response study demonstrated that these B cells were competent to move towards high concentrations (i.e., 100 and 300 ng/ml) of recombinant CXCL13 (Fig. 6c). We next assessed the ability of B lymphocytes to migrate towards supernatants obtained from stimulated or MCMV-infected microglial cell and astrocyte cultures placed in the lower wells of a chemotaxis chamber. Significant migration of B cells towards interferon (IFN)-γ-stimulated astrocyte supernatants was seen (Fig. 6d). In addition, B cells were found to move towards supernatants from MCMV-infected microglial cells, but not astrocytes (Fig. 6d). Pretreatment of the infected microglial cell supernatants with neutralizing Abs to CXCL9 and CXCL10 significantly blocked B cell migration (Fig. 6e). Neutralizing Abs to the ligand of CXCR5 (i.e., CXCL13) did not significantly block chemotaxis. The results indicated that, although these B cells were competent to move towards high concentrations of recombinant CXCL13, their migratory response to supernatants from infected microglia was primarily mediated through CXCR3 (Fig. 6e).

### B cells proliferate within the brain

**Fig. 7:**
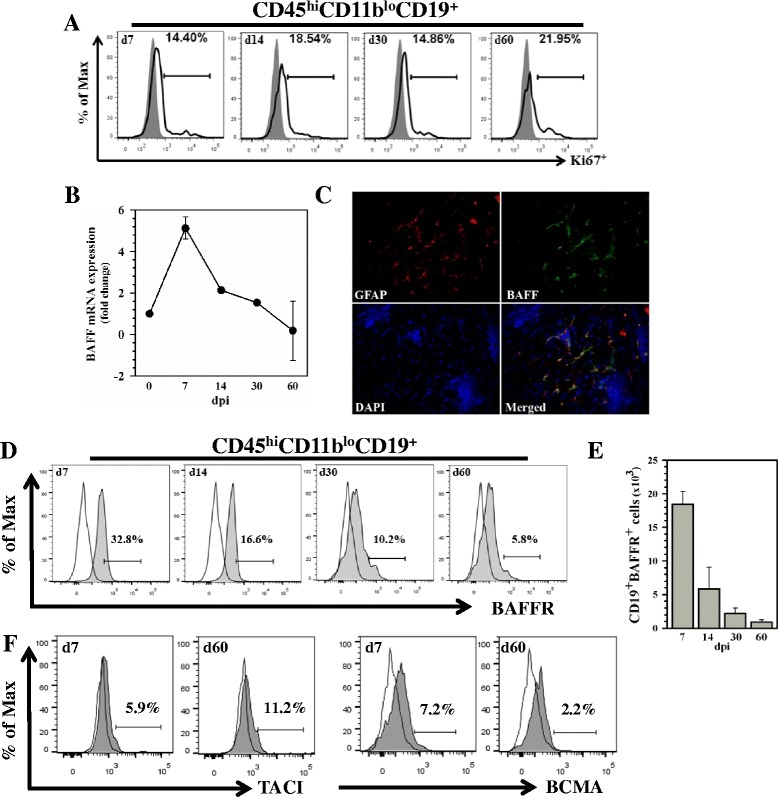
Sustained proliferation of B cells within the CNS following MCMV infection. **a** B cell proliferation within the infected brains was detected by gating on the CD45^hi^CD11b^lo^CD19^+^ population of infiltrating leukocytes followed by analysis of Ki67 (anti-Ki67-APC) expression at 7, 14, 30, and 60 increasing proportion of B cells as time. Histograms presented are representative of two independent experiments. **b** Expression of transcripts for the B cell activating factor BAFF was assessed in RNA extracted from the brains of the infected animals at the indicated time points p.i. Data presented are mean ± SEM of two separate experiments. **c** Immunohistochemical staining showing expression of BAFF protein (CD257) within the brain co-localized to GFAP-expressing astrocytes. Twenty-five-micrometer sections of the infected brain shown were obtained at 30 increasing proportion of B cells as time **d** BAFFR expression on CD19^+^ B cells within infected brains was detected by gating on the CD45^hi^CD11b^lo^CD19^+^ population of infiltrating leukocytes followed by analysis of BAFFR expression at 7, 14, 30, and 60 increasing proportion of B cells as time. Histograms presented are representative of two independent experiments. **e** Anti-BAFFR staining was used to determine the total number of B cells expressing BAFFR at the indicated time points p.i. Data (mean ± SD) are presented as absolute number of cells. **f** Anti-TACI and anti-BCMA staining was used to analyze expression of these receptors on brain-infiltrating B cells using flow cytometry at the early (7 d) and late (60 day) time points p.i. Histograms presented are representative of two independent experiments, and data presented are mean percentages from two animals per time point

BAFF and a proliferation-inducing ligand (APRIL) are well-known activating and survival factors for cells of the B-lineage and are essential for their long-term maintenance in the bone marrow [33]. Therefore, long-term maintenance of this population in the CNS also suggests the presence of these survival factors [6, 34]. We have previously reported the presence of BAFF and APRIL mRNA expression within MCMV-infected brains [7]. To determine whether B cells within the CNS were proliferating, we examined these cells for the presence of Ki67, which is present in the nuclei of proliferating or recently proliferated cells, but not in quiescent cells. Results from these studies showed that B cells within the infected brains demonstrated proliferation at early time points (14.4 %, 7 increasing proportion of B cells as time), as well as sustained proliferation persisting through the latest time point tested (21.9 %, 60 increasing proportion of B cells as time) (Fig. 7a). We confirmed the upregulation of BAFF mRNA expression within the brains of MCMV-infected mice (Fig. 7b). In addition, dual-immunofluorescence staining for BAFF protein (CD257) and GFAP demonstrated that astrocytes were a source of BAFF within the CNS at 30 increasing proportion of B cells as time (Fig. 7c). We went on to examine BAFF receptor (BAFFR) expression on CD19^+^ B cells within the infected brains by gating on the CD45^hi^CD11b^lo^CD19^+^ population of infiltrating leukocytes followed by analysis at 7, 14, 30, and 60 increasing proportion of B cells as time (Fig. 7d). The data obtained by flow cytometry were then used to calculate the total number of brain-infiltrating B cells expressing BAFFR at each time point p.i. (Fig. 7e). Additional flow cytometry experiments were performed to assess transmembrane activator and CAML interactor (TACI) and B cell maturation antigen (BCMA) on brain-infiltrating B cells at both early (7 increasing proportion of B cells as time) and late (60 increasing proportion of B cells as time) time points post-infection (Fig. 7f).

### Astrocytes promote survival and proliferation of CD19^+^ B cells in vitro

**Fig. 8:**
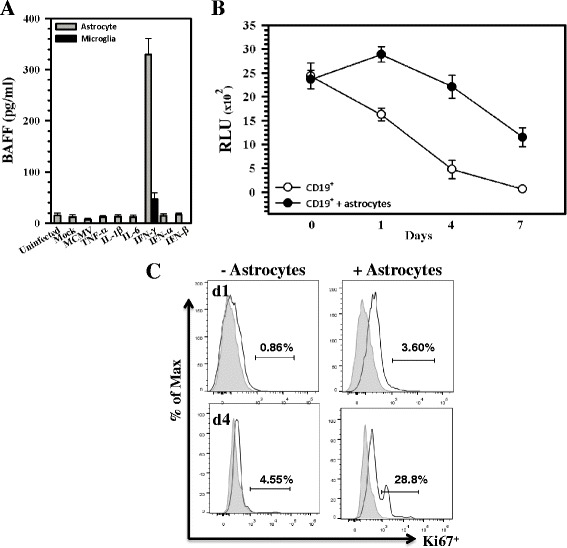
Glial cells promote survival and proliferation of CD19^+^ B cells. **a** IFN-γ treatment stimulates BAFF production by primary murine astrocytes. Astrocyte and microglial cell cultures were stimulated with MCMV or the indicated pro-inflammatory cytokine (i.e., TNF-α and IL-6, 20 ng/ml; IL-1β and IFN-γ, 10 ng/ml; IFN-α and IFN-β, 200 U/ml). BAFF levels in the culture supernatants were assessed at 48 h post-stimulation using ELISA. Pooled data (mean ± SEM) presented are derived from four independent experiments. **b** CD19^+^ B cells obtained from MCMV-primed β-actin-luciferase transgenic mice were cultured in the presence (*closed circles*) or absence (*open circles*) of primary murine astrocytes obtained from wild-type animals at a ratio of 10:1. Cultures were maintained for 0, 1, 4, and 7 days post-reconstitution, and B cell survival was assessed via luminescence. Data are presented as mean RLU (relative light units) ± SD from triplicate samples and are representative of two independent experiments. **c** Enhanced proliferation of B cells (CD19 gated) during co-culture with primary astrocytes was detected following staining with anti-Ki67-APC Abs and flow cytometry at 1 and 4 days post-reconstitution. Histograms shown are representative of two independent experiments

To investigate the capacity of glial cells to promote B cell survival, we examined primary cell cultures for BAFF production in response to stimulation with MCMV or pro-inflammatory cytokines (Fig. 8a). BAFF was found to be primarily produced by IFN-γ-stimulated astrocytes, with much lower production by microglial cells. We then isolated B cells from MCMV-primed β-actin promoter-luciferase transgenic mice (7 days post-priming) and added these cells to primary murine astrocyte cultures. Because β-actin promoter-luciferase transgenic mice ubiquitously express the luciferase enzyme, reduced luciferin intensity, as assessed using relative light units (RLU), was indicative of cellular toxicity. When co-cultured with astrocytes at a ratio of 10:1, luciferase-expressing B cells demonstrated prolonged survival over the 7-day time course when compared to CD19^+^ cells cultured alone (Fig. 8b). In addition, when the co-cultured cells were analyzed using flow cytometry, the CD19-gated population displayed enhanced proliferation over B cells without astrocytes (28.8 versus 4.55 % following 4 days of co-culture), as indicated through staining for Ki67 (Fig. 8c). When conditioned media from astrocyte cultures was used alone (i.e., no co-culture), 7.9 % of the B lymphocytes were found to proliferate at 1 day and 1.3 % were Ki67 positive at 4 days. These results indicate that astrocytes promote proliferation of B lymphocytes through a mechanism requiring cell-to-cell contact. However, clearly, astrocytes are not the only cell type which can enhance B cell survival and their survival is likely not dependent on the presence of BAFF.

## Discussion

In our previous study, we detected cells of the B-lineage which entered the brain in response to MCMV infection, produced anti-viral Abs, and persisted for at least 30 increasing proportion of B cells as time [7]. Here, we examined the kinetics of B cell recruitment into the brain mediated through chemokine production by reactive glia, along with their survival and persistence until at least 60 increasing proportion of B cells as time. Our previous study demonstrated that CD19^+^ B cells which had undergone further differentiation into CD19^−^CD38^+^CD138^+^ plasma cells were present within the brain along with CD19^+^ B cells. It is well-established that CD19^+^CD38^+^CD138^+^ plasma blasts down-regulate expression of CD19 as they fully differentiate into mature plasma cells, but at this point, it is unknown whether this CD19 down-regulation occurs prior to entry into the brain or after the cells have been recruited. Since in this study we wanted to investigate chemokine production by glial cells early during infection and its interaction with chemokine receptors during the initial recruitment of B cells into the brain, these experiments were conducted using CD19^+^ B cells.

The prominent and sustained expression of CXCR3 on infiltrating CD19 B cells over the course of infection is consistent with the high-level detection of its ligands CXCL9 and CXCL10 in brain homogenates using ELISA. The IFN-γ-inducible CXCR3 ligands CXCL9, CXCL10, and CXCL11 have previously been reported to mediate plasma blast migration in vitro [35]. Additionally, within the CNS, CXCR3-dependent plasma blast migration into the murine spinal cord during neurotropic coronavirus-induced encephalomyelitis has also been reported [22]. The results presented here demonstrate that the chemokines CXCL9 and CXCL10 were produced in response to viral infection of microglial cells, but not in response to viral infection of primary astrocytes. Likewise, B cells moved towards supernatants from MCMV-infected microglia cells, but not towards those obtained from MCMV-infected astrocytes. However, astrocytes were shown to be competent to produce these chemokines in response to stimulation with select pro-inflammatory cytokines. Previous studies investigating IFN-γ-induced CXCL9 expression in astrocytes have produced variable results. Myeloid transcription factor PU.1-mediated IFN-γ induction of CXCL9 has been reported to be limited to murine microglial cells and not astrocytes [36]. However, IFN-γ has also been reported to induce this chemokine in primary human astrocytes [37]. Differences in cell culture conditions may play a role. It appears that the cellular source of CXCL10 production depends on both viral tropism and the cytokine milieu present within the infected brain. It is likely that cytokines produced during T cell infiltration into the brain (e.g., IFN-γ expression) activate astrocytes to produce chemokines which then drive subsequent infiltration of B cells. This idea would be consistent with our previous detection of IL-21 mRNA within MCMV-infected brains at 7, but not 30, increasing proportion of B cells as time [7].

Microglial cells were not identified as a source of CXCL13 using any of the stimuli tested, but reactive astrocytes were found to be a source of this chemokine, both in vivo and in vitro. Using ELISA, we found elevated levels of CXCL13 within the brains of the MCMV-infected animals at 7 increasing proportion of B cells as time, but pretreatment of microglial cell supernatants with anti-CXCL13 Abs did not inhibit the migration of CD19^+^ B cells. Likewise, using knockout animals, CXCL13 has been reported to be dispensable for the initial recruitment of B cells to CNS inflammation induced by either Sindbis virus infection or experimental autoimmune encephalomyelitis [38]. Taken together, despite its importance in peripheral lymphoid tissue, it appears that CXCL13 may not be critical for B cell recruitment into the inflamed brain.

Glial cell-produced survival factors are well-known to play important roles in retention and survival of B lymphocyte lineage cells in the brain. BAFF [39, 40], APRIL [41, 42], and IL-6 have all been identified as critical factors for B cell differentiation and long-term survival [6]. We have previously reported the presence of BAFF and APRIL transcripts within the brain at 30 increasing proportion of B cells as time [7]. Data reported here are in agreement with previous studies using other neurotropic viruses which have localized in vivo BAFF production to astrocytes [6, 34], with production being highly inducible by IFN-γ.

Results obtained during this study demonstrate that B cells proliferated within the brain following MCMV infection until at least 60 increasing proportion of B cells as time. The proliferation of B cells in response to viral CNS infection has been reported in other models, as detected by Ki67 staining in Sindbis virus-infected mice [23]. Unlike the situation reported in multiple sclerosis [19, 43, 44], in this study, we did not observe ectopic lymphoid follicle-like structures within the infected brains. They were also not observed in the Sindbis virus model, so it is possible that they are not a consequence of viral encephalitis. Additionally, in vitro data show that astrocytes support B cell proliferation and survival when they are co-cultured at a 10:1 (B cell to astrocyte) ratio. Interestingly, astrocytes were shown to express BAFF (CD257), and brain-infiltrating B cells were shown to expresses BAFFR. Additionally, expression of TACI (CD267) was detected on 11.2 % of the brain-infiltrating B cells at 60 increasing proportion of B cells as time, along with low levels of BCMA (CD269). Like BAFFR, both of these receptors have been shown to bind BAFF and promote survival of B cell populations [45–47]. Finally, conditioned media from the astrocyte cultures alone did not potentiate B cell proliferation, indicating that B cells require cell-to-cell contact with astrocytes to maximize their proliferation.

## Conclusions

Glial cell-induced changes in the brain microenvironment promote the recruitment and survival of B-lineage cells following MCMV brain infection. Our results reveal that CXCR3 is the primary chemokine receptor on CD19^+^ B cells that persist within the brain following infection. B cell migration towards MCMV-stimulated microglial cell supernatants is largely mediated through this receptor. Correspondingly, microglial cells produce high levels of CXCL9 and CXCL10, but not CXCL13, in response to viral stimulation. Finally, reactive astrocytes were found to promote B cell survival and proliferation following MCMV infection.

## Abbreviations

ABC: avidin-biotinylated enzyme complex
ANOVA: analysis of variance
APRIL: a proliferation-inducing ligand
BAFF: B cell activating factor of the TNF family
BAFFR: BAFF receptor
BCMA: B cell maturation antigen
CNS: central nervous system
ELISA: enzyme-linked immunosorbent assay
FBS: fetal bovine serum
GFAP: glial fibrillary acidic protein
HPRT: hypoxanthine phosphoribosyl transferase
Iba1: ionized calcium-binding adaptor molecule 1
icv: intracerebroventricular
MCMV: murine cytomegalovirus
MHV: mouse hepatitis virus
MOI: multiplicity of infection
PBS: phosphate-buffered saline
RLU: relative light units
RT: room temperature
TACI: transmembrane activator and CAML interactor
TBS: Tris-buffered saline
TCID_50_: 50 % tissue culture infective dose
